# Point of Care Molecular Testing: Community-Based Rapid Next-Generation Sequencing to Support Cancer Care

**DOI:** 10.3390/curroncol29030113

**Published:** 2022-02-23

**Authors:** Brandon S. Sheffield, Andrea Beharry, Joanne Diep, Kirstin Perdrizet, Marco A. J. Iafolla, William Raskin, Shaan Dudani, Mary Anne Brett, Blerta Starova, Brian Olsen, Parneet K. Cheema

**Affiliations:** 1 Department of Laboratory Medicine, William Osler Health System, Brampton, ON L6R 3J7, Canada; andrea.beharry@williamoslerhs.ca (A.B.); joanne.diep@williamoslerhs.ca (J.D.); maryanne.brett@williamoslerhs.ca (M.A.B.); blerta.starova@williamoslerhs.ca (B.S.); brian.olsen@williamoslerhs.ca (B.O.); 2 Division of Medical Oncology, William Osler Health System, Brampton, ON L6R 3J7, Canada; kirstin.perdrizet@williamoslerhs.ca (K.P.); marco.iafolla@williamoslerhs.ca (M.A.J.I.); william.raskin@williamoslerhs.ca (W.R.); shaan.dudani@williamoslerhs.ca (S.D.); parneet.cheema@williamoslerhs.ca (P.K.C.); 3 Department of Medicine, University of Toronto, Toronto, ON M5S 1A1, Canada

**Keywords:** NGS, precision medicine, targeted therapy

## Abstract

**Purpose**: Biomarker data are critical to the delivery of precision cancer care. The average turnaround of next-generation sequencing (NGS) reports is over 2 weeks, and in-house availability is typically limited to academic centers. Lengthy turnaround times for biomarkers can adversely affect outcomes. Traditional workflows involve moving specimens through multiple facilities. This study evaluates the feasibility of rapid comprehensive NGS using the Genexus integrated sequencer and a novel streamlined workflow in a community setting. **Methods**: A retrospective chart review was performed to assess the early experience and performance characteristics of a novel approach to biomarker testing at a large community center. This approach to NGS included an automated workflow utilizing the Genexus integrated sequencer, validated for clinical use. NGS testing was further integrated within a routine immunohistochemistry (IHC) service, utilizing histotechnologists to perform technical aspects of NGS, with results reported directly by anatomic pathologists. **Results**: Between October 2020 and October 2021, 578 solid tumor samples underwent genomic profiling. **Median turnaround time for biomarker results was 3 business days** (IQR: 2–5). Four hundred eighty-one (83%) of the cases were resulted in fewer than 5 business days, and 66 (11%) of the cases were resulted simultaneously with diagnosis. Tumor types included lung cancer (310), melanoma (97), and colorectal carcinoma (68), among others. NGS testing detected key driver alterations at expected prevalence rates: lung *EGFR* (16%), *ALK* (3%), *RET* (1%), melanoma *BRAF* (43%), colorectal *RAS*/*RAF* (67%), among others. **Conclusion**: This is the first study demonstrating clinical implementation of rapid NGS. This supports the feasibility of automated comprehensive NGS performed and interpreted in parallel with diagnostic histopathology and immunohistochemistry. This novel approach to biomarker testing offers considerable advantages to clinical cancer care.

## 1. Introduction

Modern-day cancer treatment is heavily predicated on biomarker testing. Biomarker results influence all spheres of cancer treatment, but mainly systemic therapies. Biomarker-based prescription of anti-cancer therapy is the cornerstone of precision oncology treatment and represents the highest standard of care. Nonetheless, significant barriers exist in many medical practices, precluding the timely delivery of appropriate biomarker tests and matched systemic therapy. Two major barriers include the breadth and speed of testing.

The number of actionable biomarkers has steadily increased over the last decade and is expected to continue to increase over the next decade [1]. This increase in the number of clinically relevant biomarkers has led to a paradigm shift from single-marker testing to multiplex comprehensive testing, namely by next-generation gene sequencing (NGS) [2]. 

This paradigm shift is best exemplified by non-small cell lung cancer (NSCLC), where more than ten distinct biomarkers provide information that would influence systemic therapy decisions [3,4]. The practical considerations of limited tissue samples, coupled with economic considerations, make single-gene testing prohibitive, leading to the emergence of NGS as a necessary standard of care. 

**Figure 1:**
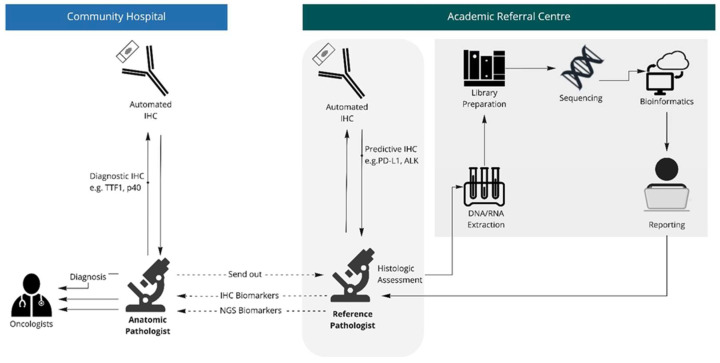
Traditional workflow. Anatomic pathologist makes diagnosis using diagnostic immunohistochemistry where applicable. Case is referred to academic reference centre where anatomic pathologist reviews histology and interprets morphologic biomarkers, with cases transferred to molecular laboratory for sequencing studies.

Access to NGS remains a barrier [5]. The technique has traditionally required subspecialized laboratory technologists, additional personnel such as bioinformaticians, and additional equipment. Thus, unlike some single-gene testing methods, such as IHC, NGS tends to occur in subspecialized facilities. These include academic hospitals, research facilities, and commercial molecular laboratories (Figure 1). 

Patients treated in publicly-funded community hospitals comprise the majority of oncology patients in many jurisdictions [6]. In this setting, access to comprehensive biomarker testing will typically require send-out testing from community hospitals to subspecialized facilities. This approach to testing is costly and creates a lengthy and convoluted pathway to gaining complete biomarker data, which in turn can lead to delays in treatment [7,8]. One recent study reported a median turnaround time of 73 days using this approach [9].

For patients with malignancies such as NSCLC, delays in test results lead to poor outcomes, with the death-rate of untreated advanced disease estimated to be 4% per week [10,11]. In-sourcing tests can significantly reduce turnaround time, resulting in faster treatments, more appropriate treatments, and fewer oncology visits [12]. 

**Figure 2:**
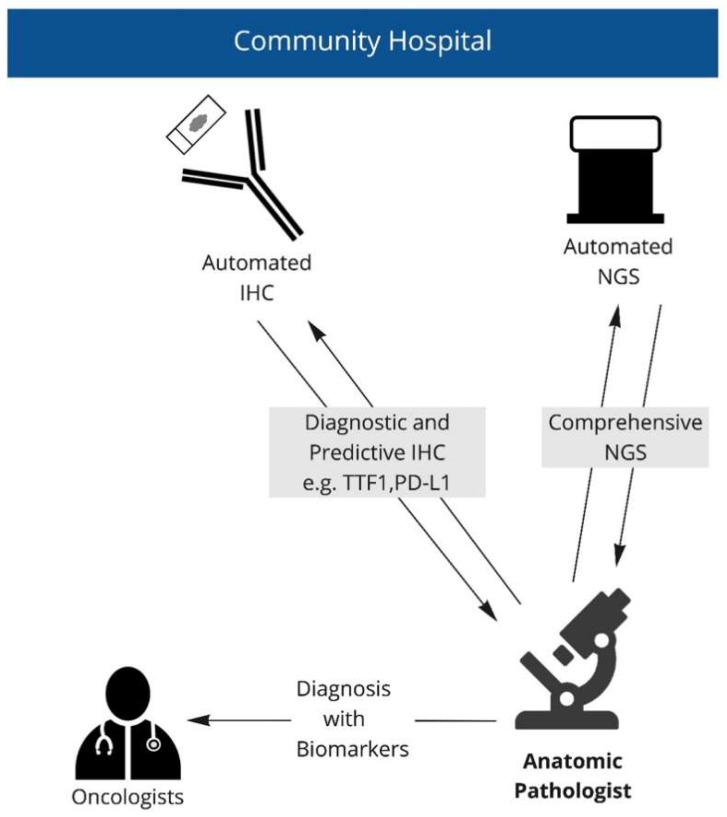
Point of care workflow. Anatomic pathologist is able to simultaneously access immunohistochemistry and next-generation sequencing. Pathologist can then report all results directly, as needed.

This report includes a review of the first cases profiled using a novel highly automated gene sequencing system [13]. The automated nature of this system enables its use directly within a diagnostic histopathology laboratory, eliminating transfers of specimens and reports. All technical aspects of NGS were performed by IHC technologists, in tandem with diagnostic and predictive IHC assays. All NGS results were interpreted and reported by anatomic pathologists and, where possible, results were integrated with IHC and other morphologic findings (Figure 2).

Here, we provide the first description of rapid comprehensive NGS in a community setting, highlighting the clinical utility of this *point of care* biomarker testing strategy.

## 2. Materials and Methods

### 2.1. Chart Review

Clinical sequencing logs were used to identify all cases undergoing clinical NGS during the study period (20 October 2020–12 October 2021). A review of the electronic medical record was performed and data was extracted, including the key dates required for the calculation of turnaround time and NGS results as reported in the patient chart. 

### 2.2. Sequencing Studies

Formalin-fixed paraffin-embedded (FFPE) samples were deparaffinized in xylene (5 min at 50 °C, washed in 100% ethanol (2 × 5 min), then air-dried for 20–45 min. Tissue digestion was performed with proteinase K (55 °C and 90 °C for 1 h each). Total nucleic acid extraction was carried out using the MagMAX FFPE DNA/RNA Ultra Kit (Thermo Fisher, Scientific, Waltham, MA, USA) and run on the Kingfisher Duoprime automated extraction system. Quantified nucleic acids were transferred to the Genexus integrated sequencer, using the Oncomine Precision Assay GX (OPA); this includes automated library preparation, sequencing, and bioinformatic analysis (Genexus Software 6.2.1, Thermo Fisher, Waltham, MA, USA). This methodology has been validated for clinical use, showing upwards of 98% concordance compared to reference methods. 

### 2.3. Specimen Handling and Work Flow

Clinical biomarker testing was initiated, either by the diagnosing pathologist (reflex), a treating oncologist, or on referred-in cases. Biomarkers consisted of NGS, as well as IHC (e.g., PD-L1 in NSCLC, MMR in CRC, among others). All NGS described in this study was performed using the OPA assay, an amplicon-based 50-gene panel including hotspot DNA analysis, copy-number assessment, and RNA fusion panel. 

Tissue microtomy, IHC, and NGS, were all performed as described, by the same group of technologists within the same division of the laboratory. All technical aspects of tissue preparation, histology, immunohistochemistry, and NGS occurred in the same physical space.

All cases were reviewed by an anatomical pathologist prior to testing for assessment of tumor content and cellularity. Molecular results were interpreted and reported by the same anatomic pathologist, in conjunction with IHC and morphologic findings, where applicable (see Figure 2). 

### 2.4. Turnaround Time Measurements

Turnaround time was defined as diagnosis date to molecular report date for reflex (pathologist-initiated) testing, request date to molecular report date for bespoke (oncologist-initiated) testing, and accession date to molecular report date for referred-in testing. A complete molecular report was defined as NGS results with applicable IHC results, visible to treating clinicians in the electronic medical record. Turnaround time was measured as *business days*, which excludes Saturdays, Sundays, and regional statutory holidays. 

### 2.5. Single Gene Testing

In silico comparison to single-gene testing in CRC included hotspots in *KRAS*, *BRAF*, and *NRAS*.

## 3. Results

**Figure 3:**
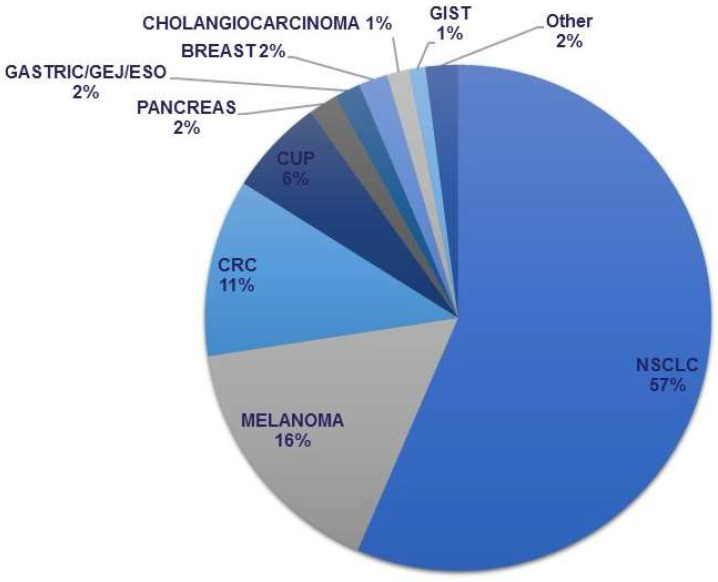
Distribution of NGS assays performed by disease site. NSCLC—Non-small cell lung carcinoma; CRC—Colorectal carcinoma; CUP—Carcinoma of unknown primary; Gastric/GEJ/ESO—Gastric, gastroesophageal junction, and esophageal carcinoma; GIST—Gastrointestinal stromal tumor.

During the study period, 578 samples underwent clinical NGS testing. These included 310 NSCLC, 97 melanoma, and 68 colorectal carcinomas, among others (Figure 3). The majority of tests were performed on in-house samples, 351 (61%), with 227 (39%) performed on referred in specimens from outside centers. Specimen type included 104 surgical resections, 411 biopsies, and 63 cytology specimens.

**Figure 4:**
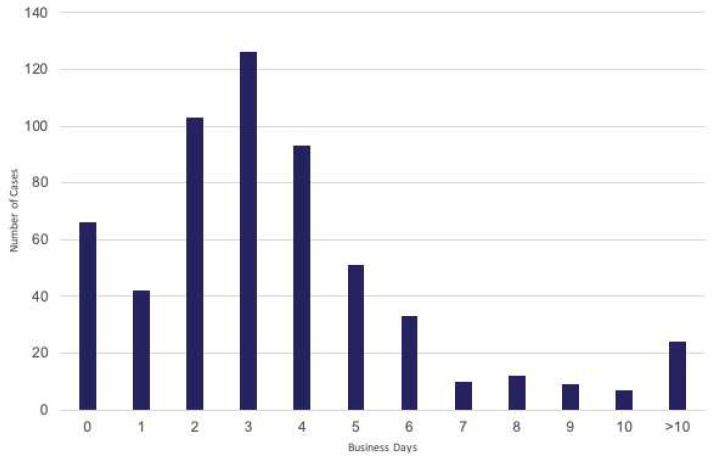
Histogram of turnaround time for all NGS reports in the study.

Median turnaround time was 3 business days for all specimens (range 0–24, IQR 2–5), and this median was observed for all cases (regardless of whether or not biomarkers were ordered reflexively at diagnosis, by an oncologist, or were referred in from outside centers). Median turnaround time was unchanged among tumor types, or across specimen classes (Figure 4).

Sixty-six cases (11%) were identified with molecular biomarker results issued simultaneously with a diagnosis (essentially unmeasurable turnaround time). Twenty-four cases (4%) were identified with a turnaround time of greater than 10 business days (Figure 4).

Of the 310 patients with NSCLC, driver mutations were detected as follows: *KRAS*, 116 (41%); *EGFR*, 51 (18%); *ALK*, 9 (3%), with additional driver alterations detected as per Table 1. Cases included reflex testing on non-squamous histologies, as well oncologist-initiated testing on squamous histologies.

Ninety-seven cases of melanoma were tested, with *BRAF* alterations detected in 42 (43%), including V600E, 22 (23%), and V600K, 16 (16%), with *NRAS* driver alterations detected in 31 (32%).

In 68 patients with colorectal carcinoma (CRC), key driver mutations were detected as follows: *KRAS*, 40 (59%); *NRAS*, 1 (1%); *BRAF*, 4 (6%); *ERBB2*, 2 (3%); and *ERBB3*, 1 (1%). Compared to in silico single-gene testing methods (including *KRAS*, *NRAS*, and *BRAF*), NGS results provided incremental utility in 12 (18%) cases, with incremental utility defined as a result that would change systemic therapy prescription. These included driver events in *ERBB2*/*3*, as well as atypical forms of *KRAS* activation, namely G12F or amplification of wild-type *KRAS*, which are not typically tested using single-gene methodologies.

## 4. Discussion

This is the first report, to our knowledge, of clinical NGS performed with a median turnaround time of 3 business days. Here, we have demonstrated the feasibility of this community-based in-house rapid NGS testing program. A median turnaround time of 3 business days was achieved for all samples, regardless of source, diagnosis, or specimen type. Delays in biomarker test results are a major concern in tumor types such as NSCLC [14]. Biomarker results are often the rate-limiting step in initiating systemic therapy, particularly in diseases such as NSCLC. Previous studies from our group have showed the turnaround time of send-out testing to be as long as 64 days, with in-sourced rapid single-gene testing delivered with a turnaround of 4 days, and that this reduction in turnaround time is associated with reductions in time to systemic therapy initiation [9,12]. This study demonstrates that comprehensive NGS can also be offered rapidly, within 3 business days. A recent study reported on the turnaround time of commercially-available plasma tests, reporting a median turnaround time of 9 days [15]. Another report describes rapid NGS-based testing on cytology samples, available within 5 days [16]. To our knowledge, this is the first reported series of cases with comprehensive biomarkers reported in as little as 3 days. 

This novel methodology has shown equal or superior performance to other NGS approaches for fusion detection [17], and here shows a detection rate of NSCLC driver mutations equivalent to those reported elsewhere [18].

This study demonstrates a novel approach to biomarker testing utilizing an automated NGS platform operated within a diagnostic histopathology service. The technological advance of automated sequencing facilitates the relocation of NGS services to within the anatomic pathology laboratory. This allows pathologists to provide biomarker data in parallel to histologic diagnoses. Sixty-one cases (11%) were resulted with NGS findings reported simultaneously to a histopathologic diagnosis, in a single document, effectively eliminating any measurable delay in biomarker results. This demonstrates the unencumbered pathway of biomarker results from tissue to oncologist, when performed and interpreted within an anatomic pathology laboratory.

## 5. Conclusions

This report describes *point of care* biomarker testing, a novel approach to provide rapid results within a community practice. The study demonstrates the feasibility of the technique, as well as the resultant turnaround time. Utilizing this approach offers considerable advantages for the clinical management of cancer patients.

## Figures and Tables

**Table 1:** Distribution of driver alterations in NSCLC samples.

Gene	Alteration	No (%)	No (%)
*KRAS*			116 (37%)
	G12C	54 (17%)	
	Other	62 (20%)	
*EGFR*			51 (16%)
	x19del	26(8%)	
	L858R	20(6%)	
	Other	5 (2%)	
*BRAF*			14 (5%)
	V600E	4 (1%)	
	Other	10 (3%)	
*ERBB2*			10 (3%)
	Mutation	7(2%)	
	Amplification	3(1%)	
*MET*			13 (4%)
	x14skip	10(3%)	
	Amplification	3(1%)	
*ALK*			9 (3%)
*ROS-1*			5 (2%)
*RET*			2 (1%)
Other			23 (7%)
None identified			66 (21%)
			310

## Data Availability

Supporting data is retained by the investigators.
